# The mental health and wellbeing of Belarusians in exile in Lithuania, Poland and Georgia

**DOI:** 10.1016/j.jmh.2025.100331

**Published:** 2025-03-30

**Authors:** Aliaksandr Kazakou, Felicity Thomas

**Affiliations:** Wellcome Centre for Cultures and Environments of Health, University of Exeter, Queen's Building, University of Exeter, EX4 4QH, UK

**Keywords:** Belarus, Exile, Persecution, Diaspora, Lithuania, Poland, Georgia

## Abstract

The large-scale international migration of Belarusian citizens is not a new phenomenon. However, it has escalated significantly since the fraudulent Presidential elections in August 2020 led to mass protests, repression, and the forced emigration and exile of between 300,000–500,000 Belarusians. This paper reports on the health and wellbeing experiences and needs of Belarusians who have migrated to Lithuania, Poland and Georgia since August 2020. Drawing on data from a survey (822 respondents), and from 60 in-depth interviews with those in exile the paper examines experiences of settlement and adjustment, challenges accessing healthcare services, migrant mental health needs and the role of diaspora groups in the provision of support. Across these themes we report how ontological insecurity and ambiguities around ‘deservingness’ have been exacerbated by the unfolding tensions and conflict across the eastern European region, with widescale detrimental impacts on Belarusian migrants’ health and wellbeing.

## 1 Introduction and background

The large-scale international migration of Belarusian citizens is not a new phenomenon. However, it has escalated significantly since the fraudulent Presidential elections in August 2020 led to mass protests, repression, and the forced emigration and exile of what is now estimated to be 300,000–500,000 people (Anon, 2024). After the election of 9th August 2020, the Central Electoral Committee announced Lukashenka the winner with over 80 % of the votes. Peaceful demonstrators in mass street rallies across the country over the following months were met with unprecedented police brutality. Thousands of people were detained, with many experiencing physical violence and torture. In 2022 alone, 5000 politically motivated detentions were registered, with the Council of Europe (Anon, 2023a) describing Belarus as ‘an immense open-air prison’, where all but the most loyal to the regime could become targets. Even the most minor ‘violations’ such as ‘likes’ of critical posts on social media or information within personal messages might now be used as evidence for criminal prosecution and detention (Anon, 2023b). Maintaining relationships with relatives and friends who remain in Belarus is increasingly difficult where contact may put them in danger. Independent media and NGOs have also been shut down or forced into exile, and opposition leaders either imprisoned or forced out of the country.

This extreme level of repression has resulted in large-scale emigration, with Poland, Lithuania and Georgia becoming major destination countries for Belarusians in exile. Whilst exact numbers are unknown, a study from July 2023 estimates the number of Belarusians in Lithuania rose from 17,769 in 2019 to 48,800 by 2022, whilst in Poland numbers rose from 25,567 in 2019 to 64,684 in 2022 (Kazakevich et al., 2023) with more recent research citing figures over 100,000 (Anon, 2023a).

Responses to this exodus within these countries can be broadly demarcated into two distinct periods, namely, from August 2020 to February 2022, and from that date to the present day (Chulitskaya et al., 2022). The time between August 2020 and the Russian invasion of Ukraine on 24th February 2022 was generally met with profound solidarity towards Belarusians opposing the Lukashenka regime, and a range of practical policy-related implications that facilitated migration and settlement. The Government of Lithuania for example, hosted the office of the widely recognized democratic leader Sviatlana Tsikhanouskaya, and simplified the process for urgent migration from Belarus despite the ongoing Covid-19 pandemic. Similarly, Poland introduced ‘Business harbour’ visas for IT-professionals and businesses to facilitate their relocation and right to work. Georgia has also been an attractive destination for Belarusians, with entitlement to a one-year visa-free stay which can be restarted simply by re-entering the country.

The second period (24th February 2022 onwards) has been characterized by the war in Ukraine in which Belarus is a co-aggressor. This has led to an intensification of repression and criminalization of those opposed to the Lukashenka regime, a sharp rise in emigration linked to a desire to avoid conscription into the Belarusian military,^1^ and a shifting of attitudes in host countries where security concerns and public opinion towards Belarusians have hardened, particularly where there is poor understanding of their predicament. Commonly associated with the Lukashenka regime, many Belarusians abroad have been unfairly linked with the aggression against Ukraine and a significant number in Poland, Lithuania and Georgia report experiencing some form of discrimination (Alampiev and Bikanov, 2022). As the Council of Europe (Anon, 2023a) have explained, ‘*the first hurdle that Belarusians in exile must clear is to gain acceptance that being Belarusian does not make them representatives or supporters of the Lukashenka regime. Perception has been a key factor contributing to the violation of the rights of the Belarusians in exile. Applying a generic treatment for Belarusians is illustrative of the absence of an individual understanding – and therefore assessment, of their situation.’*

Whilst considerable scholarly attention has focused on Belarus since the political upheaval, research has focused primarily on maintenance of political structures in exile (Jaroszewicz, Lesińska and Homel, 2022), or on comparative experiences of particular sub-groups of the pre- and post-2020 diaspora such as political activists (Korshunov and Kudrevich, 2023, Navumau, Matveieva and Gorokhova, 2024), and women (Kavalskaya, 2022). Some survey-based literature has also examined the integration experiences of Belarusians in select countries (Chulitskaya et al., 2022). This paper aims to further expand understanding by providing an in-depth qualitative insight into the health and wellbeing of the post-August 2020 diaspora in Lithuania, Poland and Georgia.

## 2 Methodology

This paper draws on research conducted between July 2022 and December 2023. Ethical approval was granted by the University of Exeter, UK. People aged >18 who left Belarus after the 9th August 2020 and who lived in Poland, Lithuania or Georgia at the time of the study were eligible to participate. In order to ensure trust in the research process, author (AK) identified the main diaspora support groups in each country and met with them to explain the purpose of the research and address any questions or concerns that they had. We then worked with them to phrase information advertising the study so that it was widely accessible and could be made available to as large a share of the diaspora as possible through their existing information channels. An online survey to generate baseline demographic data was launched through a secure platform in the three countries. The survey was open for five weeks in Poland and Lithuania (Oct-Nov 2022) and in Georgia (April-May 2023) and was available in Belarusian, Russian and English. Of the 822 responses received, 514 came from Poland, 155 from Lithuania and 153 from Georgia. Four hundred and two (402) respondents used the Belarusian version of the survey, 325 Russian and 95 the English version. Questions in the survey were divided into three sections as presented in Table 1:

**Table 1:** Survey structure.

Demographic and migration-related information	Gender, age, country of residence, reasons for leaving Belarus, reasons for choosing country of destination, time of emigration, family situation, occupation/employment situation (before and after emigration), command of local language

Role of diaspora	Role and importance of Belarusian diaspora in supporting resettlement
Health and wellbeing	Self-evaluation of health and well-being, how this was impacted by emigration, main challenges, support sought, perspectives on the future

Across the three countries, 53 % of respondents identified as women and 47 % as men (one person preferred not to say). The majority were under age 40, with 27 % aged 18–30 and 47 % aged 31–40. There were no major differences in this trend across countries, cohering with other research suggesting that the majority of those who have left Belarus since August 2020 are of working age. Sixty-two per cent of the entire sample were living with family members; of these, 65 % had one or more children. The corresponding figures for Lithuania were 66 % and 52 % respectively, in Poland they were 62 % and 71 %, and in Georgia they were 58 % and 58 %.

Prior to migration, the majority of respondents were working in mid-highly qualified posts such as teachers, medics, journalists, lawyers, academics, and engineers. A high number of IT-workers were present in the sample, ranging from 22 % in Poland to 39 % in Georgia, reflecting the ease with which this work can be transferred, and the support provided for Belarusian IT companies to relocate abroad.

Data generated from the survey does not necessarily reflect the structure of the wider Belarusian diaspora and caution must be taken in its interpretation and wider applicability. Whilst efforts were made to ensure that the survey was widely accessible, logistical and resource constraints meant that it was only available to complete online. This may have precluded participation by some potential respondents, such as older people who may feel less comfortable using online forums, and/or those without access to the internet. Citing statistical data from Lithuania and Poland, Kazakevich et al. (2023) concluded that migrants from Belarus are dominated by blue-collar workers (mainly construction and logistics) with substantial prevalence of men over women, though 2021–2022 also showed an inflow of IT-workers and other occupations. Blue-collar workers were in a minority in our survey, perhaps suggesting that they also remain “invisible” and under-represented within studies using online recruitment.

To get detailed insights into the experiences of exiled Belarusians, 60 in-depth interviews were undertaken online (20 per country). Interviewees were identified from survey respondents who had opted-in to interviews and provided contact information. A broad cross-section of participants was selected for interview based on gender and age, and broader health and wellbeing circumstances. To better understand the challenges faced, we prioritized respondents who a) assessed their/their family members’ well-being as <6 out of 10 (10 being “excellent”); b) faced significant mental health-related challenges, and/or c) had sought psychological/psychotherapeutic support. Half the interviewees were women. The distribution of interview participants across age groups was as follows (Table 2):

**Table 2:** Age distribution of the interviewees.

Age	18–24	25–30	31–35	36–40	41–45	46–50	51+
Number of interviews	7	10	17	11	6	3	6

Interviewees were informed about the management and security of their data and their rights to stop participation at any stage, and all provided informed consent. Interviews were conducted online via a secure platform. Audio recordings were translated into English, transcribed, and stored on a secure Sharepoint site. Transcribed interviews were thematically analyzed using Nvivo.

## 3 Findings

**Graph 1:**
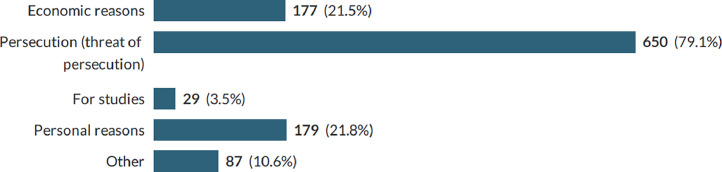
Reasons for leaving Belarus.

The survey found that *Persecution/threat of persecution* was the main reason for leaving Belarus (see Graph 1). Respondents choosing *Other* specified reasons such as fear of the war and of conscription, unwillingness to support the regime with their taxes, and being unable to see a future for themselves and their children within Belarus. While some people had time to prepare for their departure others had fled the country urgently, often within days or even hours after learning about the threat against them. Whilst 78 % felt that their wellbeing was negatively impacted by their emigration, many also recognized that leaving Belarus had improved their safety.

### 3.1 Meeting basic needs

The main challenges that Belarusians in Poland, Lithuania and Georgia identified were meeting basic needs (e.g. housing, education, economic stability), barriers to language and acculturation, and access to healthcare. Hardening public perceptions and felt diffidence around their ‘deservingness’ (Willen and Cook, 2016) as well as ontological insecurities as the prospects of returning home diminished over time cut across these themes, further exacerbating negative health and wellbeing.

#### 3.1.1 Financial needs

Attending to basic financial needs was reported as difficult, especially for those in professions not in high demand or who lacked necessary skills to enter the local job market. Liuba (age 35) for example, explained how she had initially received support in Lithuania because of her migration status. However, her inability to get a job after two years and the diminishing support available over time had negatively affected her family,

My CV has been out there for two years now. I wouldn't go study if I could find a job for an appropriate salary or within the industry I used to work in But there is no job. I need to reeducate myself which is not just learning local language but also retraining as a professional […]. A couple of months without job [will] take you down in budget dramatically. In the beginning, the Lithuanian program of support and protection of refugees was helpful, but you are in trouble if you have no job by the time the program is over.

Veronika (age 52), an established schoolteacher, had fled Belarus to avoid imprisonment. She used smugglers to help her cross the border with Lithuania illegally, fearing she would not be able to make it over the border fence erected in Poland in 2022.^2^ Unable to get a job in Lithuania, Veronika struggled financially until she secured online employment semi-legally via a Belarusian company. Having to provide for herself and her son she now works long hours to make ends meet,

I had to live on 12 Euros a week. Can you imagine? I ended up not having basic ingredients for bread, so I was making anything just to eat and not to be hungry […]. I need to support myself and my son. I am working [teaching online] in Belarus under a false name […]. Salaries paid in Belarus are not comparable to salaries paid in Euros […] My workload reaches 50 lessons a week.

Although the widespread understanding of Russian facilitated migrants’ everyday adaptation, fluency in the local language remained a basic requirement for employment, particularly in Lithuania and Georgia. Veronika's economic situation meant there was little chance to learn the local language and secure employment legally within a Lithuanian company,

Language course runs during my teaching time, and I won't sacrifice my pay for learning a language as I have to provide for my son. I have to weigh on one side opportunities for my personal integration into the local community and my child's education and wellbeing on the other side.

Challenges paying for accommodation were also problematic and exacerbated by wider political circumstances, particularly in Georgia, where a large influx of Russians following the announcement of military mobilization in September 2022 led to an almost twofold rise in rental prices. Here, even those working in the relatively well-paid IT industry reported difficulties meeting their basic needs.

The main problem is financial. There is basically no IT specialist job market […] My salary dropped, and prices went up because of the general mobilization in Russia and immigration of Russians. I really can't put any savings by. (Ilia, 36, Georgia)

Securing legal status was also a significant factor exacerbating anxiety and uncertainty. Obtaining a humanitarian visa for Lithuania or Poland for example, required evidence of prior persecution. Yet whilst the threat of persecution remains omnipresent, particularly for those actively and openly opposed to the Lukashenka regime, evidencing this threat prior to its enaction was very difficult,

There is a group of people who were not [yet] persecuted so they can't apply for humanitarian visas. Nevertheless, everyone knows them as active and engaged people. They know those people might get in trouble if they stay in Belarus. But it's like the old joke: “Come when you get killed”. “When you are arrested, we'll give you a visa” (Tatsiana, 36, Lithuania)

Those who failed to obtain a humanitarian visa in advance and those who had to leave Belarus urgently commonly had to resort to applying for asylum seeker status – a status many tried to avoid because of the lengthy processing times and, in Lithuania, the restrictions on working legally for at least the first six months. The possibility of moving on to an EU country was also restricted for Belarusians in Georgia when they faced difficulties obtaining a Schengen visa. Others reported having their application for long term residency rejected without clear reason and there are increasing reports of Belarusians who have left the host country to renew their visas being denied reentry (Anon, 2023c).

To further exacerbate challenges around legal status, Belarusian embassies ceased provision of passport services in September 2023. To arrange a new passport or undertake other legal procedures a Belarusian now has to return to Belarus, a requirement that is extremely problematic and anxiety-inducing for those who face threats of persecution. This was the case for Volha, (36) a mother of two:

When I arrived here, I applied for refugee status and one-and-half years later I'm still in suspended state. My application wasn't either rejected or accepted. I am still in a status of asylum seeker. You probably know there was a man whom they didn't let back in the country when he was doing visa run. So, I'm worried that my application will be rejected. My passport is expired, and I can't extend it via the embassy because of my criminal case [in Belarus]. There are no joint borders between Georgia and EU. If my case is rejected, I don't know what to do and where to go.

#### 3.1.2 Accessing healthcare

Many people described the challenges they had faced accessing and navigating health systems in their host country. Frustration caused by long waiting lists for state-run healthcare when many were used to paying moderate sums for private and more immediate care in Belarus was commonly cited. Olga (44) for example, explained how she felt left with little choice but to arrange crowdfunding to cover the expense of her son's surgery,

As we applied for international protection this surgery was free for us. But we were put on a waiting list. My son's condition was very painful, some fixtures he had in his leg got loose and he had to take lots of painkillers. He couldn't wait any longer, so we contacted a private hospital, and they scheduled the surgery and told us the price.

Others felt that employers took advantage of foreigners’ lack of knowledge around their healthcare entitlements. Vladimir (27) for example, explained that the type of employment contract he was encouraged to sign (*umowa o dzieło*) working in an IT-company in Poland gave him health coverage restricted only to emergency care. Dmitry (53) similarly shared his frustrations over health-related processes and his entitlements within Lithuania, having been charged for medications he had presumed would be covered by Lithuania's State Social Insurance Fund Board. Confusion around migrants’ entitlements to healthcare could also result in medical staff being unprepared to support them as Liuba described,

It was smooth on paper but people on the spot had trouble handling refugees because they didn't know the procedures. Refugees are to be registered in the database differently, under different number ID's, etc. They had never done this, or it had been done by just one person at a certain hospital who knew how to do it. But suddenly there came a lot of us and we sought medical attention, and they were overwhelmed. They didn't know how to handle us. You have all the papers, you have medical insurance from the Red Cross for a half year, but they can't find you in the database (Liuba, 35, Lithuania)

Challenges accessing and navigating health services were identified as being especially difficult for those living with mental health conditions. Ivan for example, explained his struggles to access medications, and to find a psychiatrist he could communicate with,

After 2020 I was diagnosed with a bi-polar disorder in Minsk psychiatric hospital Novinki. I am still undergoing treatment and take medications. I took three medications, I've run out of two and am running out of the third one. I face a problem because I don't know how to make an appointment with a Russian-speaking psychiatrist in Poland. I seem to defer this appointment as I simply don't have the strength to deal with this issue.

Used to a system of treatment including medication and psychotherapy as well as support with administrative tasks within Belarus, Katerina (32) also explained how she had struggled to access comparable help in Lithuania. Without support to navigate the complex systems and processes she was required to engage in to gain employment, accommodation and medical support, she described her situation as a ‘vicious circle’ in which she, like Ivan, felt continually exhausted and weakened by the bureaucracy she was required to confront.

### 3.2 Mental wellbeing

#### 3.2.1 Ontological insecurity

Challenges attending to basic needs and securing legal status had a range of negative impacts on the mental health and wellbeing of study participants. Many explained how this was exacerbated by an escalating sense of ontological insecurity, experiences of discrimination, ambiguities around ‘deservingness,’ and the ongoing trauma of persecution, compounded by limited access to healthcare.

Study participants who had left Belarus in the initial months after the repression began in 2020 explained that early optimism that Lukashenka's regime would not endure meant that many people felt hesitant to invest their efforts into starting a new life abroad. Reflecting on the growing realization that exile was becoming a long-term prospect, Nadziezhda explained the uncertainties and associated anxieties her family now faced,

I personally had a feeling, I was sure that we would come back, that all this is a very temporary thing. Then, when those signals began that they [Belarusian authorities] could pass a law to confiscate properties my husband decided to sell the flat in order [to get] at least some money in case something somewhere happens. And once we were having no home where to come back to… It's been unclear, in short, unclear… And still I have a feeling that possibly we will come back, albeit unclear where to, or maybe we will stay here, but here it is also unclear. In short, there is no certainty at all. And I can tell you that out of my circle practically everyone is in this condition of uncertainty. Everyone acts differently, but the feeling of uncertainty stays on for practically everyone. (Nadziezhda, 38, Poland)

Yulia felt similarly, explaining that despite help from psychotherapists and from diaspora support groups over the past two years, she could not accept her status in exile,

I want to go home. I find it difficult in principle to come to terms with things that I don't accept in life and let go of the past. I am strongly attached to my past and I don't agree, I don't understand, I refuse to understand. Yes, I refuse to understand, that's the key point. I don't understand it (Yulia, 28, Poland)

Importantly, this uncertainty also played out in the ways that study participants sought to make sense of and narrate their situation. Although the majority had left Belarus because of threats and/or experiences of persecution, the corresponding vocabulary for these circumstances was avoided and often replaced by far more euphemistic phrasing. Rather than being *expelled* from their home country for example, people spoke of being *moved* (*пераехаць* in Belarusian, *переехать* in Russian) – a more neutral term denoting relocation from one place to another within a country or abroad. The term *рэлакацыя*/*релокация* - a direct adaptation of the English *relocation* was also used. Hence, those who ‘relocated’ were referred to as *relocants* (*рэлакант*/*релокант*), or even as ‘*expats*’. It is possible that the use of such neutral terminology instead of terms like *migrants* or *refugees* is a sign of (potentially unconscious) self-protective behavior to detract from the very real ontological insecurities experienced in everyday life.

#### 3.2.2 Discrimination and deservingness

Lukashenka's support for and involvement in Putin's invasion of Ukraine was felt to have influenced a widespread hardening of public attitudes towards Belarusians. In a recent survey, 39 % of Belarusians in Georgia, 31 % in Poland and 16 % in Lithuania reported experiencing some sort of discrimination or enmity based on their origin (Alampiev and Bikanov, 2022). In our study, almost all participants were aware of instances of discrimination against Belarusians in their host country, although few reported experiencing this first-hand. One exception was Olga, a single mother in Poland, who described the experiences of her 12 year old son,

I think it's [discrimination] a common problem. I had to personally deal with it – and the problem is still ongoing. It's expressed in prejudice, and excluding behaviour against children. My child was in an integration class. Children were not feeling comfortable, they were criticized for speaking Russian between themselves. Social media was full of comments – “you go to your Lukashenka”, “Russian language is evil and shouldn't be spoken” […] I wasn't speaking Polish well and wasn't able to protect my son (Olga, 33, Poland)

Like many others, Nika (35) explained how much of this discrimination was exacerbated by general ignorance regarding the situation in Belarus,

Truly, I met so many Poles who don't know at all what is going on in Belarus. I am happy for them, but they displayed total ignorance. They don't understand why we move […] I had a visitor from social services whose job is to inspect the living conditions of the migrants in Poland. She still wasn't aware why we are moving to this country. She was in shock listening to my stories.

Nina, (41) Lithuania, described how the shift in attitudes towards those from Belarus had deeply unsettled the legitimacy of their previously unquestioned status. At the start of the war, she had offered the spare room in her apartment to people from Ukraine. Explaining how her offer was snubbed once they learnt she was from Belarus, she described the ongoing pressure on Belarusians to justify their status,

[having the apartment rejected] was a traumatic experience for me, [but] of course I can understand. You carry on explaining and explaining that it's not the Belarusian people, that we are actually occupied by the Russians and so on. It's kind of hard and these negative reactions from Ukrainian side affect us, because before that the local people helped Belarusians with open heart. Now when they see you and your documents, they start asking whether you are pro-Putin or pro-democracy. So, you need to clarify all the time that you're pro-democratic Belarusian.

These circumstances also influenced perceptions of self-deservingness, with a widespread feeling that it was difficult and even inappropriate and shameful to openly claim their suffering. Nika described this reluctance as a ‘national trait’, explaining that Belarusians did not like to seek help when there were others who needed it more,

I tend not to ask for help, because I don't feel that I need it more if I compare myself to other people in harder circumstances. I think it's our national trait when we constantly feel we do not deserve something good and feel that other people need it more (Nika, 35, Poland).

This was exacerbated when people compared their plight to that of the Ukrainians now living alongside them in their host country. Kaciaryna (32) for example, explained that whilst psychological support was increasingly available through Ukrainian staff, she did not feel morally entitled or able to access this,

According to my doctor, psychotherapy should help […] My brain was fucked up and I suffered a lot psychologically when the war happened […] I was ashamed speaking to therapists from Ukraine, I just couldn't compare my problems with those devastations people suffered in Ukraine. Even if Ukrainian psychologists are ready to work with Belarusians, I don't feel comfortable.

#### 3.2.3 Mental health and trauma in exile

Study participants spoke of the different degrees of stress, anxiety and trauma they experienced in exile. Over 40 % of survey respondents reported seeking help for their mental health. Of these, 45 % (*n* = 150) had approached psychologists or psychotherapists for support. Whilst this indicates high rates of poor mental health and wellbeing, ongoing stigma around mental health problems (Anon, 2023d) means this is almost certainly a considerable underestimate. Interviews with people who described their poor mental health also revealed how a lack of resources, language barriers and/or the extremity of their condition further limited their capacity to seek professional support.

Many study participants had left Belarus due to threats and escalating fears around persecution. Several described the stress endured trying to explain to their children the gravity of their situation and the need to flee the very real threat of persecution for what were often very minor or seemingly farcical ‘violations’ within Belarus. Talking about the difficulties her young son faced understanding the situation, Irina, for example, described how household tensions were incited after their move to Poland,

There was a very difficult period of adaptation over the first three months, he was locking himself in a room, barricading the doors, calling me names, refused to get out of bed at all, refused to eat, did not talk to me. (Irina, 37, Poland)

Loneliness was commonly cited as a problem, particularly in the early months following arrival in a host country. Whilst often overlooked within discussions around migrant mental health, homesickness can also be a cause of significant and sustained stress (Rosner et al., 2022) with research finding that separation from family can contribute to increased rates of depression, PTSD, anxiety, and poor quality of life (Liddell et al., 2022). For some Belarusians who had not only left their loved ones behind but also left them incarcerated in the state's detention and torture facilities, trauma was significantly amplified. Aliaksandr (38) for example, explained how he had fled to Ukraine after he was warned he was ‘being hunted by the KGB’. Following the arrest of his colleagues, he entered a state of depression and described how his ‘inability to take direct part in the process to free them or to end the war, is very traumatic for me.’

Aliaksandr's case also emphasized how the trauma of exile had been amplified for a sub-set of Belarusians who had initially fled to Ukraine, where they went through period of adaptation and settlement only to then face upheaval again following the Russian invasion. Aleksandra also described this accumulation of stress and trauma,

Starting from 2020, that depressing environment, it was all superimposing together. In Belarus it was all negative, negative and depressing. We came to Ukraine, lived there a year, a bit more than half a year being rather relaxed, and the war broke out and then all that follows - the relocations, and then my boyfriend went away, I found myself alone in a foreign country not knowing what to do. It all came together, and it became very hard (Aleksandra, 25 Poland)

Many participants described the ongoing trauma of persecution. Some described their individual experiences of detention and torture and the associated witnessing of state-led brutality. Yauheni for example, was tortured at Akrestsina, the infamous detention centre run by the Minsk Executive Committee's Main Internal Affairs Directorate. Now living in Georgia, he was unable to afford professional help to cope with his mental health issues. He reflected on his ongoing trauma and the nature of the violence he was exposed to,

In my view, this is a post-traumatic syndrome that stays with you for the rest of your life. It's something experienced by people who got raped or something like that. It continues to follow you. Maybe if one day justice prevails in the most favourable conditions for us, then seeing the rule of law and justice, the victim could breathe a sigh of relief. Otherwise, the trauma remains. That inhumane attitude, I don't even know how to call it, which a person was subjected to in Akrestsina, a person who otherwise had a normal way of life, such person is not even able to explain that such things are even possible […] to understand the nature of it is just beyond comprehension (Yauheni, 38, Georgia)

Yana similarly explained the impact that the ongoing trauma of persecution and exile had on her wellbeing. She experienced arrest by the KGB and fled the country after her release. Asked why she did not seek professional help after leaving Belarus she described the intense magnitude of her fear,

That's not an easy question I would say. I didn't want to leave my place at all. I was very scared even to go shopping. I thought no one would understand me anywhere, everything seemed to be confusing and scary […] First few weeks I had a feeling that someone was following me. I understood that my feelings were irrational, but I still couldn't help but be scared […] When you are fearing to go to a store other than the nearest one you are not going to see psychologist for sure because you think you wouldn't be understood (Yana, 21)

Others explained how the possibilities of seeking psychological help were also bound up with the broader regional political situation. Whilst some such as Kaciaryna (32) felt unable to seek support from Ukrainian professionals, others, such as Aliaksandr explained his hesitancy to seek support in Georgia where there was a high likelihood that a psychotherapist would be Russian – a situation he felt would undermine his mental health. Furthermore, and overarching these concerns was widespread feeling that a lack of understanding of the complexity of the Belarusian context meant that very few psychotherapists would ever be able to provide effective support, as Elena (64) for example, explained,

She (psychologist) doesn't understand the Belarusian situation. The first appointment was brutal because I had to relive all the trauma which I had been through in Belarus, in order to explain my situation to the therapist. During the second appointment the specialist tried all known methods, reasons to explain to me why I can't go back. She kept telling me one phrase: “You need to forget that you had a home.” I understand that she's maybe not very good at speaking Russian. But when she tells me that I need to draw a line and forget about home and start living a new life - I listened to her advice and then answered that it would be better for me to forget that I had a psychotherapist.

Vicarious and collective trauma through exposure to media pictures of traumatic events is well-recognised (Holman et al., 2020, Hopwood and Schutte, 2017) and was reported in this study to transmit through media coverage of the violent events surrounding protests and state-led repression. Unlike more traditional forms of media, social media, e.g. Telegram-channels, were felt to be especially impactful due to lower censorship allowing for publication of more disturbing content. Recent research on collective trauma amongst Belarusians found that almost all surveyed were affected by the witnessing of police violence against protesters on videos published online (Korshunau, 2024). Fewer than 10 % of those sampled in that study did not know anyone who had received some form of legal punishment (from short term arrest to criminal charges) for their anti-Lukashenka stance and almost a third knew someone imprisoned (ibid).

Whilst fear and trauma of persecution was ongoing for many we interviewed, there was also recognition that the relative safety of their host country provided opportunities for healing. Katsiaryna, for example, had worked as a journalist for a media organisation labelled as extremist by Lukashenka's regime. Already receiving psychotherapy sessions before she left Belarus it was not until moving to Georgia that her well-being started to improve,

I can write freely without the fear of being persecuted. It was very hard psychologically to start with, but it's certainly better now. I sleep better and I am not scared of the noises on the staircases by my door. As I understood, my emotional state affected my physical wellbeing including hormonal disbalance. I suffered loss of hair due to stress. Having moved to this country, my stress level diminished, this country is definitely free compared to Belarus. (Katsiaryna, 20)

### 3.3 Diaspora, support, and belonging

Diaspora and diaspora groups constituted an important source of support for Belarusians in exile. Fifty-eight per cent of survey participants felt they had benefited from the Belarusian diaspora through individual connections and/or via diaspora support groups. Whilst the percentage of those who had approached individual Belarusians for support was comparable across the three countries (66 % in Poland, 63 % in Lithuania, 67 % in Georgia) the figures differed considerably for those seeking support from diaspora groups, at 60 %, 57 % and 17 % respectively. Such a low percentage in Georgia is likely to be explained by the more recent influx of migrants and the associated under-development of practical support local Belarusian groups are currently able to offer.

The most frequent issues respondents sought assistance with correlate with the main settlement and adaptation challenges discussed above. Support provided ranged from the sharing of information and advice on social media to practical help with legal issues from qualified lawyers, provision of financial and material support (including temporary accommodation), language courses, seminars to support adaptation, e.g. on starting a new business, educational courses for children, and leisure-based activities. The majority of study respondents were satisfied with the help they received from these sources, although some felt more support was needed in relation to employment, securing of legal entitlements, help for children and adolescents and psychological/psychotherapeutic support. Others felt that a lack of advocacy from political diaspora structures and poor coordination between different groups could detract from their potential and mean that people needing support did not always receive it. One interviewee working in a human rights organization in Lithuania for example, explained how a lack of awareness raising and coordination left some people isolated and vulnerable,

The problem of the Belarusian organization is lack of systematic approach. Some do this, others do that, and people who happened to learn about help come for it - those who don't miss it. One of my acquaintances, who fled Belarus […] spent a year living in Vilnius in a complete information vacuum. I realised that when I accidentally met him, and he told me his story. For example, he didn't know about the [Telegram] chat “Belarusians in Vilnius” […] he was paid illegally by cash. I was really shocked a person wasn't aware of simple things.

While 58 % of the survey respondents felt they benefited from the wider Belarusian diaspora, 80 % said that the diaspora was important to them, suggesting it is valued beyond its capacity to provide practical support. The political uprising of 2020–21 witnessed what has been described as unprecedented solidarity amongst (anti-regime) Belarusians, with grassroots self-organising and the rise of ‘peoplehood’ and national identity within a society that had previously felt quite disconnected (Gabowitsch, 2021; Kazharski, 2021; Korosteleva and Petrova, 2023; Kulakevich, 2020; Bekus, 2021). The new Belarusian diaspora appears to have preserved, at least to an extent, the level of unity showed at the time of political mobilization in 2020 with study respondents frequently describing its value through positive phrasing such as a “feeling of being home”, “community of like-minded people”, “community of our people”, and its existence helping them to “retain language and culture” and uphold “Belarusian identity”. Others explained how the very existence of the diaspora felt important in terms of psychological well-being and reassurance (“if it wasn't for diaspora, I wouldn't feel comfortable here” - Katsiaryna, 20, Georgia) and reminding them that they were not alone (“my connection with diaspora practically helped me to survive and not to go into depression” - Alena, 58, Georgia).

Yet whilst the diaspora plays a vital role in supporting wellbeing, it is worth noting that the strength of its existence may also be delaying settlement and integration into host communities. Tatsiana (36) for example, described how easy it was to live in a kind of Belarusian bubble within Lithuania, and how the tools (Telegram chats/channels) used to mobilise people facilitated this,

I come across Lithuanians only when I need to when executing some paperwork in a bank, resolving bookkeeping matters, shopping, coffee shops. I wouldn't call it integration. Some contacts are pretty random. Our communication is mostly around Belarusian people. There are a lot of Belarusians, we even found a Belarusian plumber in a local chat.

By the time of this study, anxieties around long term exile had started taking hold. However, and as previously discussed, many of those who had been fuelled by initial optimism around the imminent fall of Lukashenka's regime experienced an understandable hesitancy to expend large amounts of energy integrating into host communities. Several years on, however, they face a dilemma over the role of the diaspora and the possibility that they are creating unhelpful barriers within the societies that they now live within. Kirill (32) for example, fled to Poland believing that he would return home within a year or two. He now lives in an apartment block accommodating a large number of Belarusians who brought with them the practice of *dvaravyya chaty* (Telegram ‘yard chats’) that take place within buildings/neighbourhoods, and described how this restricted opportunities for integration,

In our house chat we have 100 subscribers, neighbours from other houses would like to join. So, we have a conflict – should we keep it for our building or include them and make it a district chat. At the forefront of these debates there is a question of why we created these chats, what's the purpose. Should we create a ghetto, or diaspora? Or should we integrate in Polish culture, stop supporting this native language chat and join Polish people social chats via Facebook, not Telegram.

Interestingly, whilst 80 % of survey respondents found the diaspora to be important, this dropped to only 70 % amongst those who had left Belarus in 2020. Further research is needed to understand this, but it is possible that those who fled earlier are now making more concerted efforts to integrate into their host communities and gradually weakening their connection with the Belarusian diaspora as the possibilities of an imminent return home diminish.

## 4 Discussion

The political violence and unrest witnessed in Belarus since August 2020 has resulted in the mass emigration of 300,000–500,000 Belarusians to other countries. As this paper has demonstrated, many of those who left Belarus for neighbouring countries continue to face everyday challenges relating to their legal and financial security, as well as extreme trauma and ongoing fear relating to experiences of, and/or the possibilities of arrest and persecution. As time has gone on and the chances of a safe return to Belarus under the Lukashenka regime diminish, people are being forced to confront the practical ramifications and mental anguish of their increasingly clear status as exiles. Whilst not all of those interviewed wished to openly acknowledge or describe themselves in this way, many explained that they were starting to reflect more on their need to reach beyond the Belarusian community if they were to attempt to integrate better into host societies.

However, at the same time as the likelihood of returning safely to Belarus has reduced, those who fled repression are now caught up in the ongoing fall-out of wider regional conflict. The initial political and popular support for Belarusians in neighbouring countries is universally felt to have waned since Russia's invasion of Ukraine, with widespread misunderstanding which homogenizes Belarusian aggression further undermining people's already poor mental wellbeing and felt deservingness within host countries. As Paul Galle, Rapporteur to the Council of Europe's Committee on Migration, Refugees and Displaced Persons stated in the Luxembourg Solutions conference in June 2024, ‘the plight of the Belarusians in exile is in danger of becoming a forgotten or misunderstood tragedy’ without awareness raising and the implementation of practical solutions within host nations.

Many of the recommendations put forward in the Luxembourg Solutions would, if implemented, go some way to improving the mental health and wellbeing of Belarusians in exile through, for example, amending legislation around legal entry, visa status, and economic opportunities, as well as improving access to education, and promoting Belarusian cultural identity. However, given the enormous levels of political violence, persecution, torture and trauma experienced amongst this population, it is likely that a more systematic approach to support these more serious mental health needs is also required. Some relatively small-scale initiatives do exist - both within the Belarusian diaspora and internationally - to provide targeted mental health support that recognizes the particular context of trauma and persecution faced by those fleeing the Lukashenka regime. However, such initiatives are currently extremely resource constrained, and only able to offer very piecemeal and short-term support to a small number of people. Whilst such interventions are welcomed, it is clear that large numbers of people require far more specialized and sustained levels of mental health support. It is vital therefore that countries wishing to support Belarusian people in exile work not only to remove the many current obstacles to the legal and practical support needed to be able to settle and thrive, but also need to proactively work alongside the diaspora and with the many mental health care professionals (including many who are themselves Belarusian) who live within their borders to identify how this more specialist and context informed support can be resourced, sustained and effectively delivered.

## CRediT authorship contribution statement

**Aliaksandr Kazakou:** Writing – original draft, Project administration, Methodology, Investigation, Formal analysis, Data curation. **Felicity Thomas:** Writing – review & editing, Supervision, Project administration, Methodology, Funding acquisition, Formal analysis, Data curation, Conceptualization.

## Declaration of competing interest

The authors declare that they have no known competing financial interests or personal relationships that could have appeared to influence the work reported in this paper.

## Footnotes

1 Notably, 15777 Belarusians entered Georgia within just three weeks after the start of Russian invasion of Ukraine; almost ten times more than the same period in 2019 (Новости-Грузия 2022).

2 Poland began work on an 18-foot steel wall aimed at blocking the passage of illegal migrants following Lukashenka's actions to encourage illegal migration into the European Union in the late summer of 2021. During this time, Poland did not close its border crossings with Belarus or stop granting asylum. However, it is likely that the wall deterred some Belarusians who may otherwise have gone to Poland.
